# Simulations of Magnetohemodynamics in Stenosed Arteries in Diabetic or Anemic Models

**DOI:** 10.1155/2016/8123930

**Published:** 2016-02-25

**Authors:** Aiman Alshare, Bourhan Tashtoush

**Affiliations:** ^1^Mechanical Engineering Department, German Jordanian University, Amman 11180, Jordan; ^2^Mechanical Engineering Department, Jordan University of Science and Technology, Irbid 22110, Jordan

## Abstract

Pulsatile flow simulations of non-Newtonian blood flow in an axisymmetric multistenosed artery, subjected to a static magnetic field, are performed using FLUENT. The influence of artery size and magnetic field intensity on transient wall shear stress, mean shear stress, and pressure drop is investigated. Three different types of blood, namely, healthy, diabetic, and anemic are considered. It is found that using Newtonian viscosity model of blood in contrast to Carreau model underestimates the pressure drop and wall shear stress by nearly 34% and 40%, respectively. In addition, it is found that using a magnetic field increases the pressure drop by 15%. Generally, doubling the artery diameter reduces the wall shear stress approximately by 1.6 times. Also increasing the stenosis level from moderate to severe results in reduction of the shear stress by 1.6 times. Furthermore, doubling the diameter of moderately stenosed artery results in nearly 3-fold decrease in pressure drop. It is also found that diabetic blood results in higher shear stress and greater pressure drop in comparison to healthy blood, whereas anemic blood has a decreasing effect on both wall shear stress and pressure drop in comparison to healthy blood.

## 1. Introduction

Atherosclerosis is an accumulation of cholesterol-laden plaque in arterial walls that causes a narrowing or stenosis and a loss of elasticity in the arteries at various sites. The diseased arteries often result in heart attacks and strokes both of which are leaders in human mortality. The major cause of stroke is blood vessel blockage or plaque rupture. Narrowing of an arterial lumen tends to occur in regions of disturbed flow and oscillating wall shear stress (WSS). Tan et al. [1] linked the growth, progression, and structure of plaque in a 70% carotid symmetric stenosis at rupture to the oscillating wall shear stresses using pulsatile transitional simulations. Considering axially asymmetric stenosis and Newtonian fluid model Gao et al. [2] found that the Womersley number has a great influence on the vortex generation and the WSS distribution and to a lesser extent on the Reynolds number. Grinberg et al. [3] analyzed the flow in stenosed carotid artery using three-dimensional transient model and a simplified two-dimensional slice, since the latter is more appropriate as clinical tool. Their results revealed that regions of unsteady laminar flow characterize the state of the flow and a subregion of turbulence, starting downstream of the stenosis and extending about five to six centimeters farther downstream. The flow in the subregion is found to laminarize as the Reynolds number is decreased. Pulsatile flow of Newtonian blood through stenosed porous medium with periodic body acceleration under the influence of a uniform transverse magnetic field was studied by Das and Saha [4]. The study proposed that varying the effect of the magnetic field in, for example, clinical magnetotherapy, can regulate the volumetric flow rate.

Numerical simulations have been proposed by Ku et al. [5] as a method for predicting changes in flow distributions and patterns from surgical bypass procedure. The simulations' results were temporally and spatially averaged and compared against measurements obtained using magnetic resonance imaging (MRI) techniques for a phantom model of a stenotic vessel with a bypass graft under conditions suitable for surgical planning purposes. The maximum error in the computed volumetric flow rates was 6% of the measured values; also an excellent qualitative agreement was obtained for the cross-sectional velocity profiles in both magnitude and shape. Karmonik et al. [6] employed CFD simulations for three vascular pathologies of the human aorta with patient-specific geometries and inflow boundary conditions.

The potential for obtaining information helpful in therapeutic decision making was demonstrated by analyzing selected hemodynamic parameters such as blood flow pathlines, wall shear stresses, dynamics pressures, blood flow velocities, and flow particle residence times. Sankaranarayanan et al. [7] transient model of aorta-coronary bypass graft showed that maximum perfusion of the occluded artery occurs during middiastole, and the maximum wall shear stress variation is observed around the distal anastomotic region. Pulsatile flow through a stenosed artery using Casson model for blood was investigated by Sankar and Lee [8]. Their results showed that intensifying the magnetic field leads to a decrease in flow rate and an increase in skin friction. Casson, Newtonian, and the hybrid blood constitutive models were used in the pulsatile flow simulations in human carotid bifurcation and were reported by Fan et al. [9]. The results showed that Newtonian and the hybrid model gave similar distributions of WSS and axial velocity. The study suggested the inadequacy of using the Casson model for the entire flow field and its use should be limited to flow regions where the shear rate is low.

Using Large Eddy Simulation (LES) in a three-dimensional geometry of an arterial stenosis Molla and Paul [10] performed pulsatile transition-to-turbulent non-Newtonian blood flow with various blood viscosity models. The effects of the various viscosity models are investigated in terms of the shear rate, poststenotic recirculation zone, mean shear stress, mean pressure, and turbulent kinetic energy. Pulsatile flow in an arterial stenosis and in the presence of a transverse magnetic field has been studied by Ikbal et al. [11]. Results have shown that both the vessel wall flexibility and Reynolds number affect the flow characteristics and the development of recirculation zones upstream of the constricted site, while the magnetic field causes reduction of the flow rate. In addition, many researchers reported an association between blood viscosity changes and human cardiovascular diseases such as hypertension, spasm, and thromboembolism [12, 13]. Kuke et al. showed that blood viscosity has large effect on duration of cerebral ischemia and reperfusion [14]. Many studies also focused on several factors that influence the blood viscosity [15–19].

Moreover, apparent viscosity of human blood was found to be significantly influenced by magnetic field. Haik and coworkers reported a 30% decrease in blood flow rate when subjected to a high magnetic field of 10 tesla, which indicated an increase in the apparent viscosity [20]. Yadav et al. reported a 30% decrease in blood flow rate and a 45% increase in the apparent blood viscosity in a capillary tube model subjected to a magnetic field of 0.002 tesla [21]. Bali and Awasthi [22] investigated the effect of external magnetic field on blood flow in stenosed artery and considered the viscosity of blood as radial coordinate dependent. Assuming blood a Newtonian fluid and accounting for ferrohydrodynamics and magnetohydrodynamics effects, Tzirzilakis, in his steady flow model, predicted a generation of stronger vortices downstream of the stenosis throat when magnetic field was applied [23]. However, blood is a non-Newtonian fluid and was modeled as a generalized Power law fluid by Ikbal et al. [11] to investigate atherosclerotic arteries with mathematical models that represent non-Newtonian flow of blood through a stenosed artery in the presence of a transverse magnetic field. On the contrary, Li and Huang [24] predicted suppression in the vortex formation downstream of the stenosis when blood was assumed to be a Power law fluid rather than a Newtonian fluid. Kenjereš [25] presented a numerical analysis of blood flow in realistic arteries subjected to strong nonuniform magnetic field. Habibi and Ghasemi [26] investigated the effect of a magnetic field on the volume concentration of magnetic nanoparticles of a non-Newtonian blood. Kwon et al. [27] studied the effect of blood viscosity on oxygen transport in residual stenosed artery. Sankar et al. [28] numerically investigated pulsatile laminar blood flow through a mild symmetric stenosis treating blood as Hershcel-Bulkley fluid concluded that velocity decreased with increasing Hartmann number and amplitude of the flow. Tashtoush and Magableh [29] studied the effect of magnetic field on blood flow through multistenosed artery treating the blood as Newtonian fluid, whereas Alshare et al. [30] investigated steady flow in moderate and sever stenosed arteries in the presence of magnetic field and treating the blood as non-Newtonian by applying the Carreau viscosity model.

In this work, a pulsatile two-dimensional analysis of blood flow with variable viscosity through arteries with multiple stenosis in the presence of magnetic field is investigated. In order to establish the applicability of the investigated model in the realm of blood rheology, the blood viscosity will be considered for two cases, namely, when blood viscosity is constant and when blood viscosity is allowed to vary with shear rate according to the Carreau-Yasuda model. In addition, three blood types are investigated using the Carreau model of viscosity, namely, healthy, diabetic, and anemic blood. The governing equations with their corresponding boundary conditions are solved using CFD.

## 2. Method

### 2.1. Mathematical Modeling and Problem Formulation

Blood is considered as an electrically conducting fluid; when subjected to a magnetic field an electromagnetic force is produced and an electrical current flows as a result. The problem consists of the solution of the transient governing momentum Navier-Stokes equations and the electrical Maxwell's relations for the magnetic field.

The current density *J* is expressed by

(1) $$\begin{array}{c} J=\sigma \left(\;E+V\times B\;\right), \end{array}$$

where *E* is the electrical field intensity, *σ* is the electrical conductivity, and *V* is the velocity vector. In the momentum equation, the electromagnetic force, *F*_*m*_, is defined as follows:

(2) $$\begin{array}{c} F_{m}=J\times B=\sigma \left(\;E+V\times B\;\right)\times B. \end{array}$$

**Figure 1:**
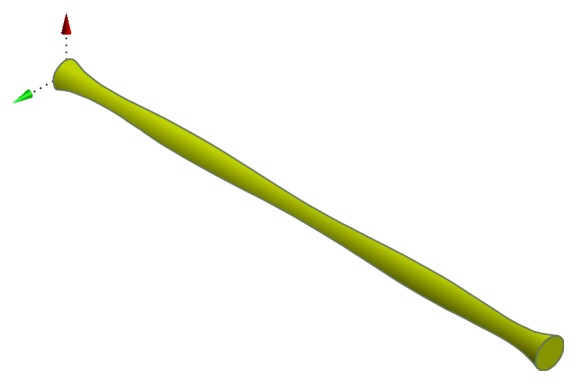
The geometry of artery with multistenosed sections.

The multistenosis artery under consideration is assumed to be a rigid cylindrical tube containing a homogeneous non-Newtonian fluid representing the blood. Use the cylindrical coordinate system (*r*, *θ*, *z*), where the *z*-axis is taken along the axis of the artery, while *r* is taken along the radial direction. Since the flow is assumed to be axisymmetric, the angle, *θ*, effect is neglected. The geometry of the stenosis in the arterial lumen is shown in Figure 1 and is taken as follows [31]:

(3) Rz=Ro1−α1.48z−0.7398z2+0.1485z3−0.013955z4+0.0006145z5−0.000010243z6.

*R*(*z*) indicates the radius of the artery in the region of interest and *R*_*o*_ is the radius of the normal artery; *L* is the length of the stenosis and *α* is the degree of stenosis.

The continuity, momentum, and energy equations governing the flow under consideration, in cylindrical coordinates, are as follows.

*Continuity Equation*. Consider

(4) $$\begin{array}{c} \frac{\partial v_{r}}{\partial r}+\frac{v_{r}}{r}+\frac{\partial v_{z}}{\partial z}=0. \end{array}$$

*Radial Momentum Equation*. Consider

(5) ρ∂vr∂t+vr∂vr∂r+vz∂vr∂z=−∂p∂r+μ∂∂r1r∂rvr∂r+∂2vr∂z2=0.

*Axial Momentum Equation*. Consider

(6) ρ∂vz∂t+vr∂vz∂r+vz∂vz∂z=−∂p∂z+μ1r∂∂rr∂vz∂r+∂2vz∂z2−σB2vz=0.

The boundary conditions associated with the governing equations for the problem are as follows:

  No slip condition at the artery wall:
  (7) $$\begin{array}{c} v_{r}=v_{z}=0\;\text{at }r=R_{o}. \end{array}$$
  At the center line, the blood velocity is finite:
  (8) $$\begin{array}{c} \frac{\partial v_{z}}{\partial r}=0\;\text{at }r=0. \end{array}$$

### 2.2. Method of Solution

The dimensional-form of the governing equations along with the boundary conditions, (4)–(8), is solved in terms of the primitive variables. The nondimensional equations and the relevant dimensionless numbers in this study such as Reynolds and Hartman numbers were not utilized since, in non-Newtonian model, the dynamic viscosity, which is dependent on the shear rate, enters both dimensionless numbers.

### 2.3. Non-Newtonian Fluid Model

The Carreau-Yasuda model is used in hemodynamical simulations given as follows [32, 33]:

(9) $$\begin{array}{c} \mu \left(\;\dot{\gamma }\;\right)=\mu_{\infty }+\left(\;\mu_{o}-\mu_{\infty }\;\right){\left[\;1+{\left(\;\lambda \dot{\gamma }\;\right)}^{a}\;\right]}^{\left(n-1\right)/a}, \end{array}$$

where *a*, *n*, and *λ* are empirical constants; $\dot{\gamma }$ is a scalar measure of the rate of deformation tensor [34]. In this study, the values obtained by Gijsen et al. [32] are used and given as follows:

(10) μo=22×10−3 Pa s,μ∞=2.2×10−3 Pa s,a=0.644,n=0.392,λ=0.11 s.

Three blood types are investigated using the Carreau model of viscosity, namely, healthy, diabetic, and anemic blood. This is accomplished by varying viscosity parameters in the model to correspond to different hematocrit level in the blood that represents these conditions. Following experimental data of Kwon et al. [27] a blood with hematocrit count of 45% is considered healthy blood, whereas a diabetic and an anemic patient's blood hematocrit count is 65% and 25%, respectively. Summarized in Table 1 are the hematocrit count and the corresponding viscosity parameters.

### 2.4. Computational Model and Validation

**Figure 2:**
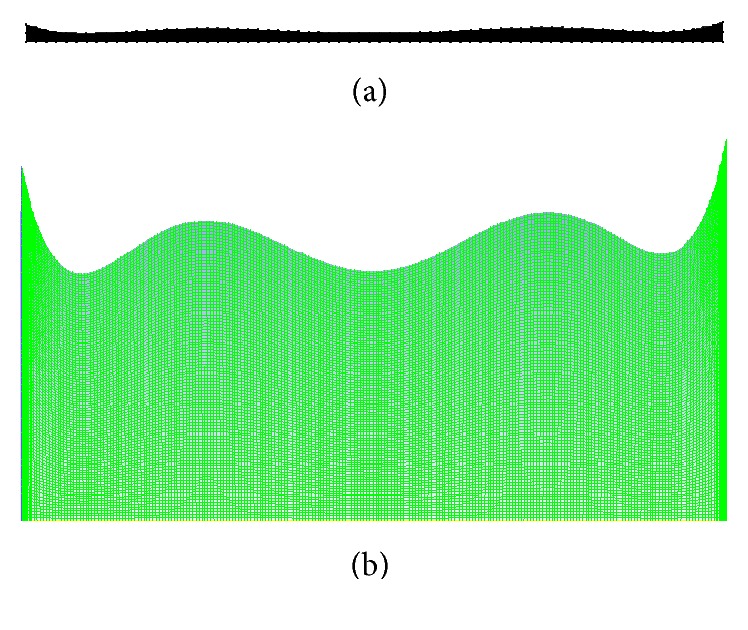
An axisymmetric section of a 10 mm diameter of stenosed artery showing the computational mesh (a) and a figure (b) scaled up 20 times in the *r*-direction for illustration clarity.

A commercially available package ANSYS Work Bench FLUENT 15.0 [35] which is based on Finite Volume Method (FVM) was used. The axisymmetric computational domain of the multistenosis artery geometry given by (3) was constructed and meshed using design modeler and mesh tool, respectively. Temporal discretization was by the second-order implicit backward Euler scheme [36]. The second order upwind scheme was utilized in the spatial discretization. Pressure-velocity decoupling was handled using the SIMPLE algorithm [37]. The inlet wave form of the cardiac cycle is monitored. The residuals for the convergence were monitored and solutions are converged when the mass and velocity residuals were less than 10^−7^. A nonuniform mesh of 130 × 130 shown in Figure 2 was used in all the simulations.

**Figure 3:**
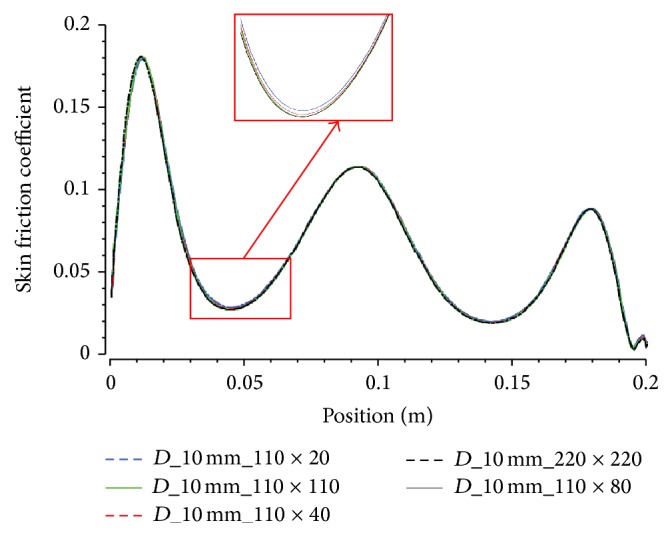
Show the local skin friction factor using 5 different grid sizes.

A progression of increasing mesh sizes was tested to ensure mesh independent results as shown by the average skin friction factor, *C*_*f*_, values given in Table 2 for the three artery sizes considered in the study. The variation of local skin friction factor with various grid sizes is also illustrated in Figure 3.

**Figure 4:**
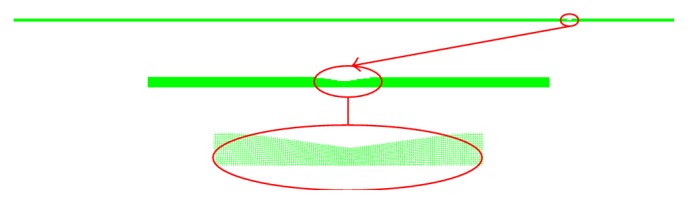
The computational domain used for the validation of the model with experimental data of Tiari et al. [38]. Actual domain (top) and stenosis section (bottom) are shown zoomed in for clarity.

In order to validate the numerical results a computational domain of an axisymmetric artery with a single stenosis, shown in Figure 4, similar to the experimentally tested stenosis geometry by Tiari et al. [38] is considered.

**Figure 5:**
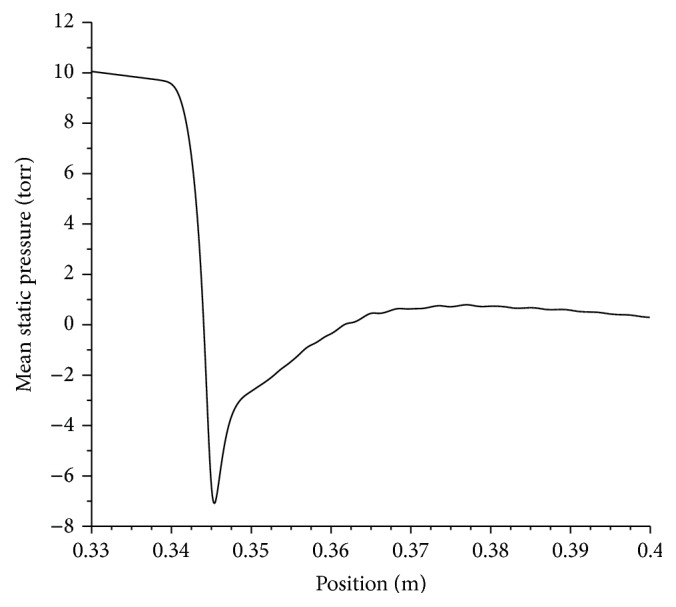
Pressure drop across a single stenosis (54%) in a 3.12 mm artery. Pulsatile wave from experiment [38] is employed.

First, the domain and the used grid were tested without stenosis and the pressure drop in the planar artery region beyond the entrance length where the flow is fully developed is checked with the Poiseuille law, the error in the pressure drop is found to be less than 0.1%. Then a single stenosis with a severity level of 54% is employed. The inlet pulsatile volumetric flow rate wave form used in the experiment which has a peak value of 9 mL/s is utilized. The resulting mean pressure drop across the stenosis is nearly 9.5 mmHg as shown in Figure 5.

It is generally in good agreement with experimentally obtained value of 7.6 mmHg. The deviation between the results could be attributed to a number of factors including tube compliance, curvature uncertainty, and experimental uncertainties. The computational model assumes a rigid artery wall while in the experiments a compliant tube with elastic properties similar to that of a coronary artery is used.

### 2.5. Inlet Boundary Condition and Additional Source Terms

**Figure 6:**
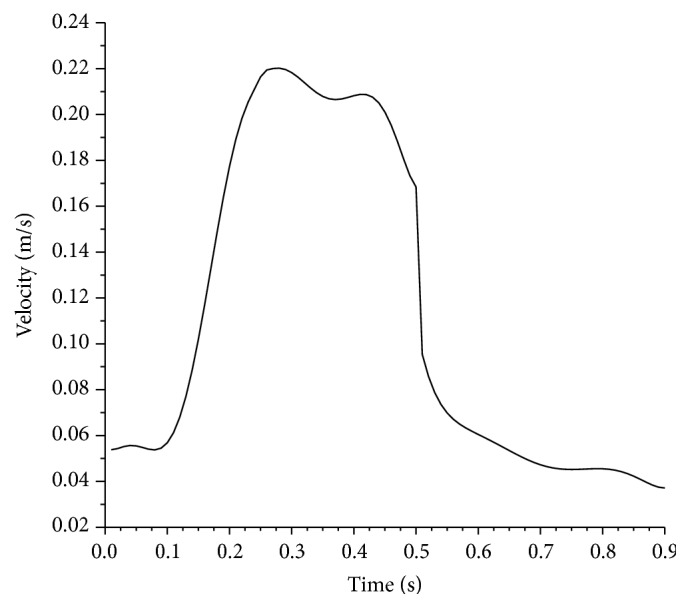
Pulsatile velocity wave form.

The Womersley method was used to define the pulsatile velocity waveform [39, 40] at the inlet of the computational domain. The transient and patient-specific inflow velocity waveform following [1] is illustrated in Figure 6.

The physiological waveform of the heart was segmented into three portions. Each segment is fitted with a high-order polynomial function over a subinterval of time. The fitted waveform was coupled to the computational model through a user-defined function (UDF) capability offered by FLUENT. The blood is investigated using the constant viscosity model and non-Newtonian fluid model. The latter is introduced in the model using UDF.

The imposed constant magnetic field that appears in the axial momentum equation as an additional source term and represents the Lorentz force was handled with UDF.

Blood was treated as a non-Newtonian fluid with a density of 1050 kg/m^3^ and a viscosity governed by (9). This was compared with the case where blood was assumed to be Newtonian with a viscosity of 3.0 m Pa·s. Non-Newtonian viscosity was incorporated into FLUENT computational model through UDF. Each simulation is carried out using 3 cardiac cycles. Each cycle is one second, which is subdivided into 90 time steps.

## 3. Results and Discussion

Hemodynamics in arteries has an unsteady character caused by the cyclic pumping of the heart. Both blood flow and pressure are pulsatile. The interplay between oscillatory inertia forces and viscous forces in flowing blood is characterized by the Womersley number, a nondimensional parameter relevant to this type of flow. Figure 6 illustrates the unsteady inlet waveform employed in this study. However, the non-Newtonian viscosity along with pulsatile velocity which is a function of the strain rate resulting in changing viscosity not only spatially but also temporally makes it meaningless to compute a Womersley number under such conditions. In the following discussion, we will consider the geometric, viscosity, and magnetic parameters effects on the mean wall shear stress and mean pressure drop.

**Figure 7:**
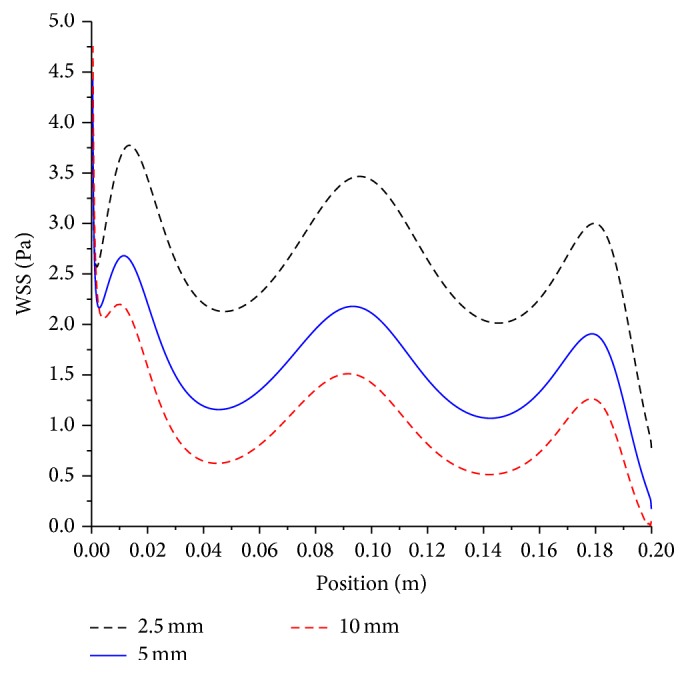
Effect of artery size on mean wall shear stress; artery diameter, 2.5 mm, 5 mm, and 10 mm; magnetic field, 4 tesla; non-Newtonian viscosity, moderate stenosis condition.

**Figure 8:**
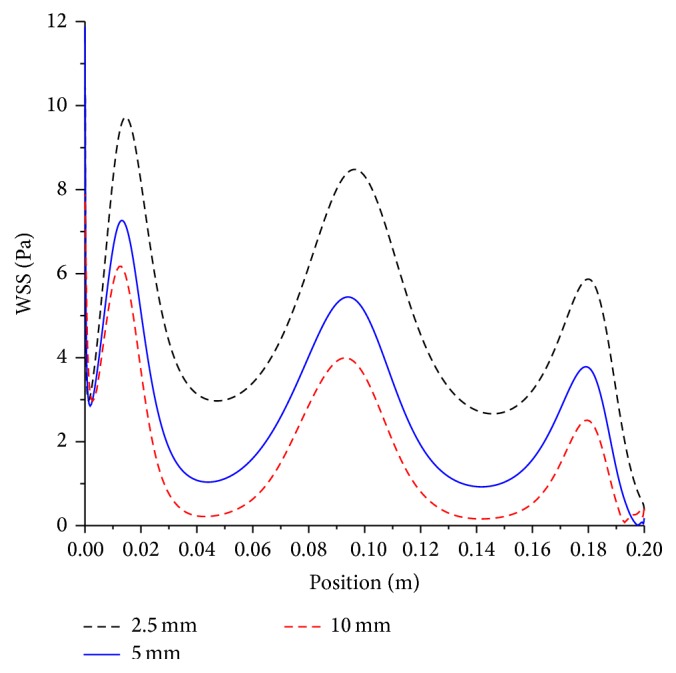
Effect of artery size on mean wall shear stress; artery diameter, 2.5 mm, 5 mm, and 10 mm; magnetic field, 4 tesla; non-Newtonian viscosity, severe stenosis condition.

The effect of the artery size on the mean axial shear stress for both moderate and severe stenoses is shown in Figures 7 and 8, respectively.

Artery diameters of 2.5, 5, and 10 mm are investigated. As the artery diameter decreases by half, the mean wall shear stress increases by nearly 1.6 times, whereas if the diameter is reduced by 4-fold, the mean shear stress increases by 2.6 times. Generally, this implies that doubling the artery diameter will decrease the shear stress by approximately 1.6 times for both moderate and severe stenosis. When the level of stenosis increases from moderate to severe, the mean wall shear stress increases by 1.6 times on the average for all artery sizes. To nullify the effect of increased level of severity on shear stress, the artery size has to be doubled.

**Figure 9:**
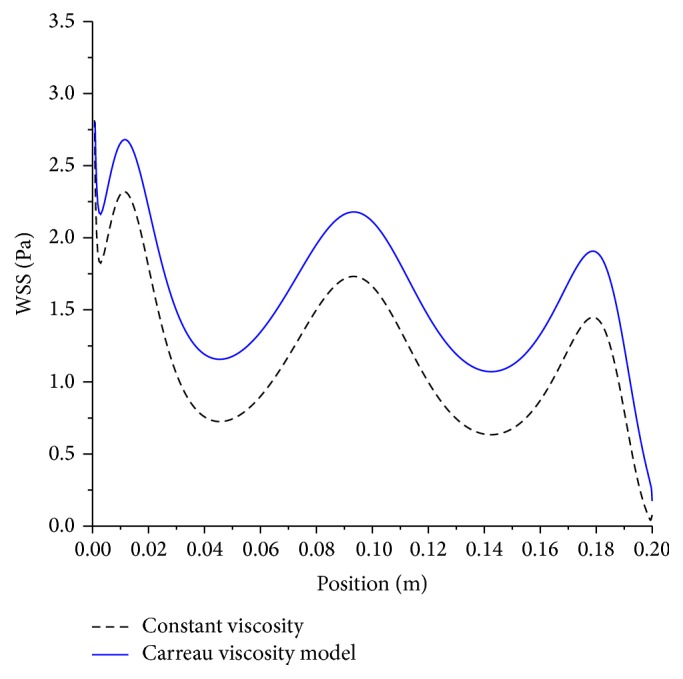
Effect of viscosity on mean wall shear stress; artery size, 5 mm; magnetic field, 4 tesla; and Newtonian and non-Newtonian viscosity models.

Modeling the blood as Newtonian fluid in contrast to the non-Newtonian, namely, Carreau-Yasuda results in underestimating the mean wall shear stress by about 40% as illustrated in Figure 9.

Modeling the blood, as non-Newtonian fluid, is essential as the diameter of arteries decreases and the shear thinning is exhibited which allows the red blood cells to squeeze through the small capillaries. When the shear rate is low the non-Newtonian character also shows up and the red blood cells agglomerate into larger particles.

**Figure 10:**
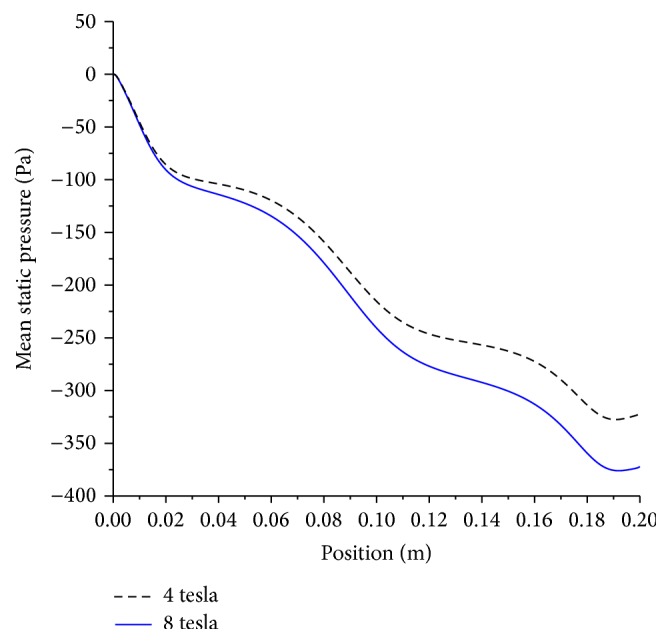
Effect of magnetic field on mean pressure drop; artery size, 5 mm; magnetic field, 4 and 8 tesla; non-Newtonian, moderate stenosis.

The effect of doubling the magnetic field from 4 to 8 tesla increases the pressure drop by nearly 15% as given by Figure 10, but it shows marginal influence on the wall shear stress.

**Figure 11:**
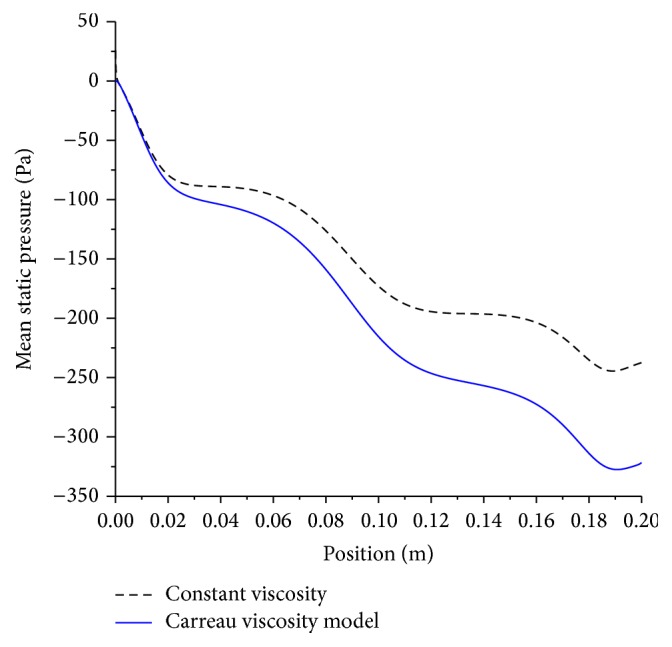
Effect of viscosity on mean pressure drop; artery size, 5 mm; magnetic field, 4 tesla; and Newtonian and non-Newtonian viscosity models.

However, using Newtonian fluid model of blood in contrast to non-Newtonian Carreau viscosity model underestimates the pressure nearly by 34% as can be inferred from Figure 11.

**Figure 12:**
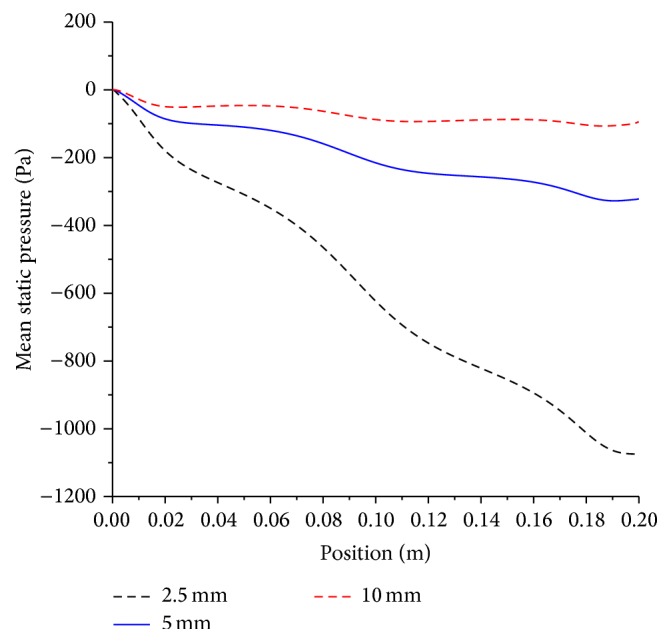
Effect of artery size on mean pressure drop; artery diameter, 2.5 mm, 5 mm, and 10 mm; magnetic field, 4 tesla; non-Newtonian viscosity, moderate stenosis condition.

**Figure 13:**
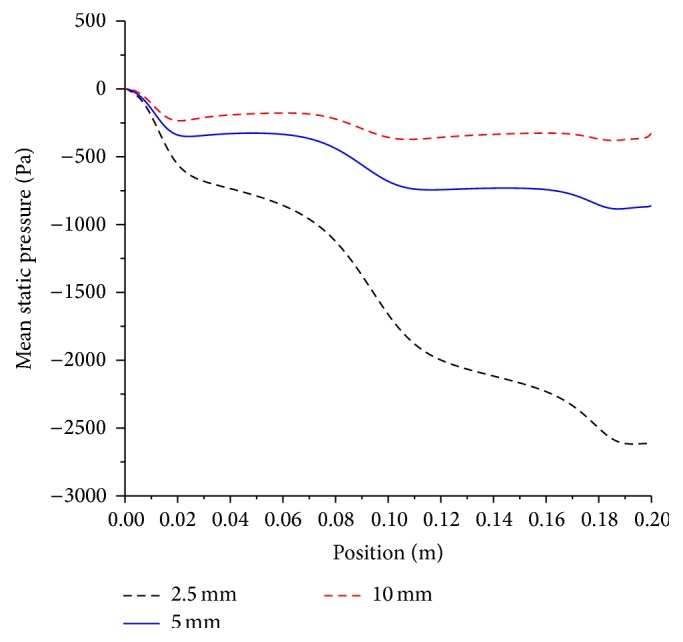
Effect of artery size on mean pressure drop; artery diameter, 2.5 mm, 5 mm, and 10 mm; magnetic field, 4 tesla; non-Newtonian viscosity, severe stenosis condition.

The effect of the artery diameter and stenosis severity on pressure drop is very large as shown in Figures 12 and 13.

Doubling the diameter of stenosed artery results in approximately 3-fold decrease in pressure drop. The pressure drop trends, which do not follow the Poiseuille law, are indicative of the significance of the role the inertial term effect on the pressure drop. The stenosis sections present location of favorable pressure gradient, as evident by three local “humps” on each curve. The implication of this local acceleration and in particular when the stenosis is of a severe level is detrimental. It would assist in dislodging and mobilization of fresh thrombus material, which can be heighted during catheterization procedure. On the other hand, increasing the stenosis from moderate to the severe results in approximately 3-fold increase in pressure drop, which can be attributed to decreased effective area available for the flow and the aforementioned physiological adverse effects will hold again. Therefore, the effect of increasing stenosis from moderate to severe has the same effect as halving the artery diameter on the increase in pressure drop. The conjugated effect that is increasing the stenosis level from moderate to severe in conjunction of halving the diameter will result in a 9-fold increase in pressure drop. A moderate stenosis in 2.5, 5, and 10 mm arteries results in a pressure drop of 8, 2.5, and 1 mmHg, respectively. Meanwhile, a severe stenosis in the same sizes has a pressure drop of 20, 6.7, and 2.6 mmHg.

**Figure 14:**
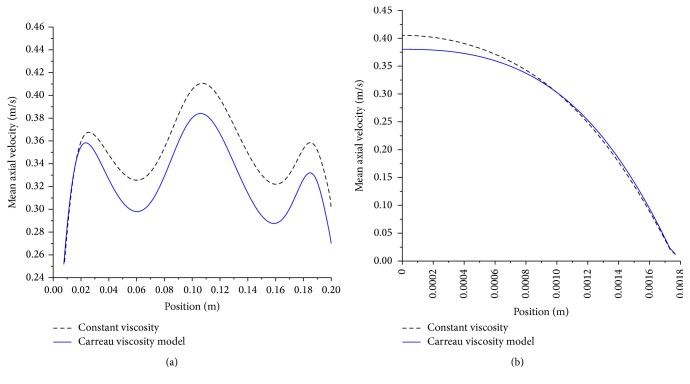
(a) Effect of viscosity on axial velocity profile (artery centerline); artery size, 5 mm; magnetic field, 4 tesla; Newtonian and non-Newtonian, moderate stenosis, pulsatile flow. (b) Effect of viscosity on axial velocity profile (cross section in middle of artery); artery size, 5 mm; magnetic field, 4 tesla; Newtonian and non-Newtonian, pulsatile flow.

In order to further assess local effects of the viscosity model, the axial velocity along the artery centerline and at a cross section in the middle of the artery is plotted in Figure 14.

It is apparent that using the Carreau viscosity model will decrease the local velocity by nearly 7%.

**Figure 15:**
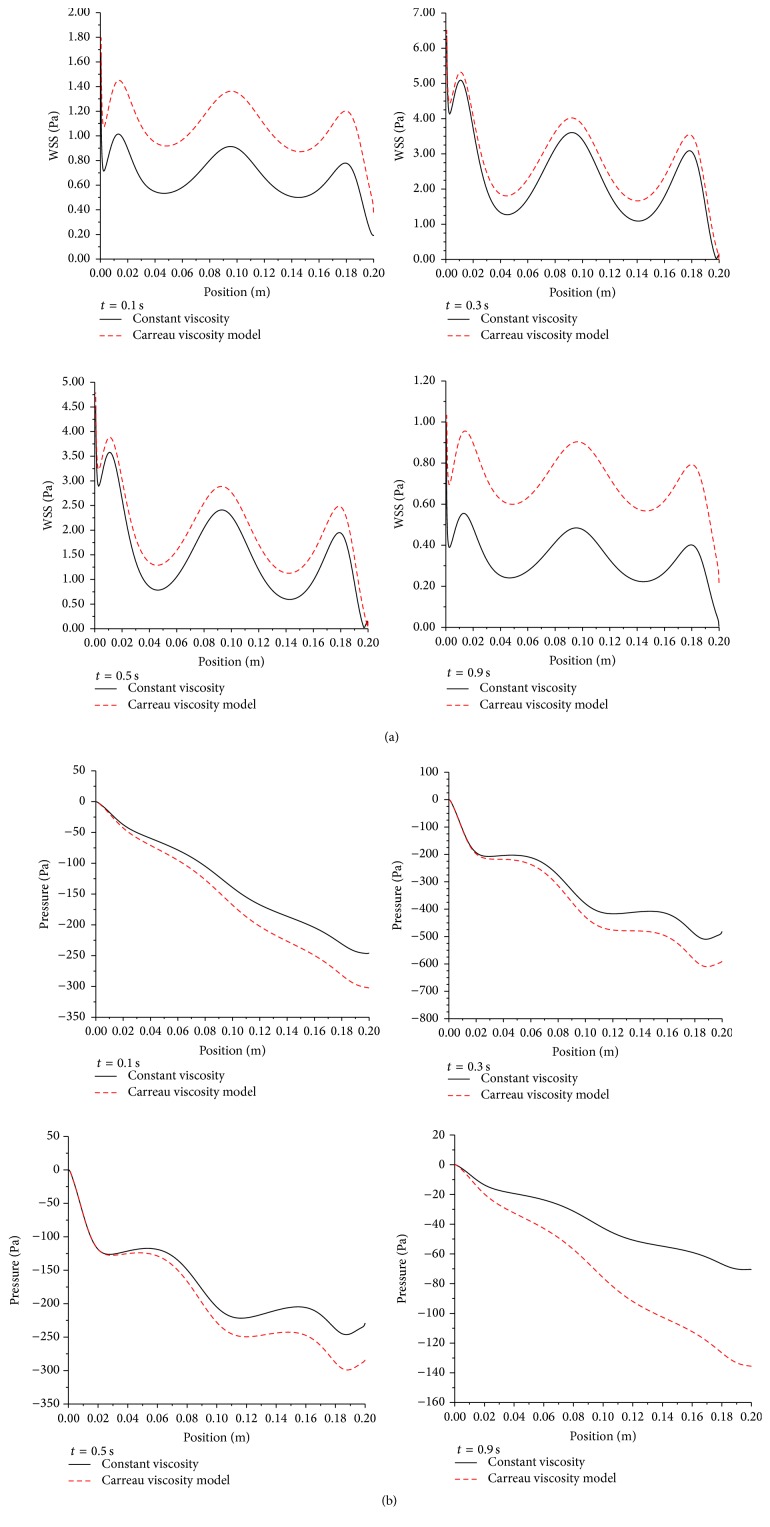
(a) Instantaneous wall shear stress (Pa) at different time steps in the cardiac cycle. Artery diameter 5 mm with moderate stenosis. (b) Instantaneous pressure drop (Pa) at different time steps in the cardiac cycle. Artery diameter of 5 mm with moderate stenosis.

Instantaneous values of wall shear stress and pressure drop are plotted in Figure 15 at selected sequential time steps of the cardiac cycle.

Due to the nature of the cardiac cycle illustrated by the pulsated velocity profile in Figure 6, the instantaneous wall shear stress increases with the corresponding increase in velocity but also it increases due to local acceleration in the stenosed sections as depicted by peak to valley sequence along the profiles. In addition, the pressure drop increases with increasing velocity and decreases with increasing local acceleration due to favorable pressure gradient in the stenosed zones.

**Figure 16:**
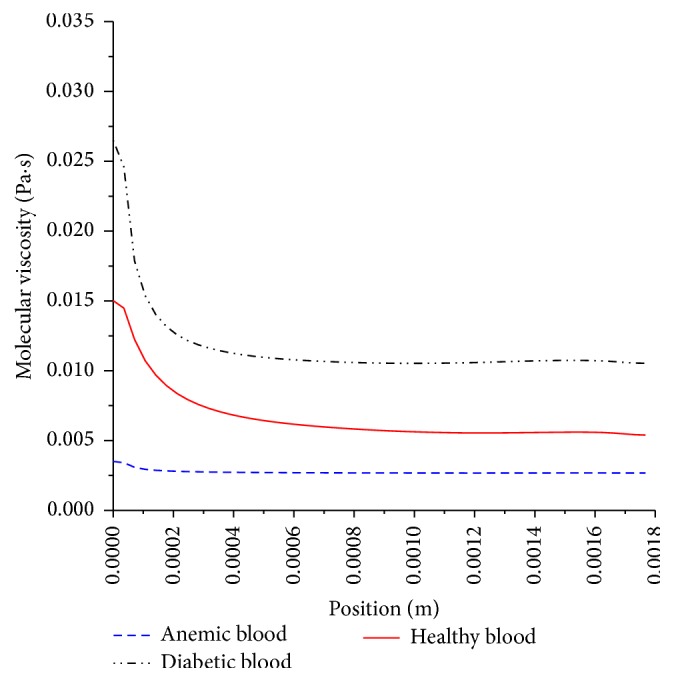
Carreau viscosity for anemic, diabetic, and healthy blood type, taken at cross section in the middle of the artery.

Simulations of pulsatile flow through a 5 mm diameter artery using Carreau viscosity models according to the parameters given in Table 1 is carried out.  Figure 16 shows a snapshot of the local viscosity for the three blood types at a midsection in the stenosed artery.

**Figure 17:**
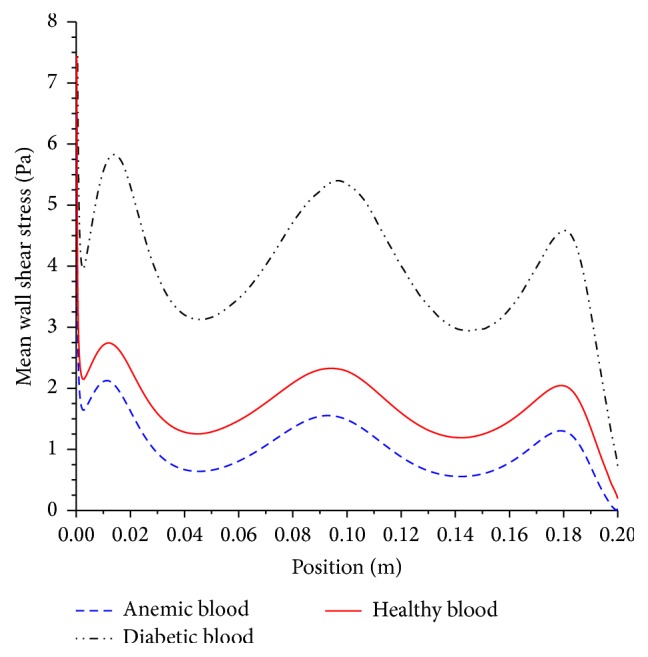
Mean wall shear stress. Artery diameter of 5 mm with moderate stenosis, using anemic, diabetic, and healthy blood types.

It can be seen that a diabetic blood has higher viscosity, due to higher volume percentage of red blood cell or “thicker” blood. This translates to higher resistance or greater shear stress at the artery wall of diabetic blood in comparison to healthy blood as shown in Figure 17.

**Figure 18:**
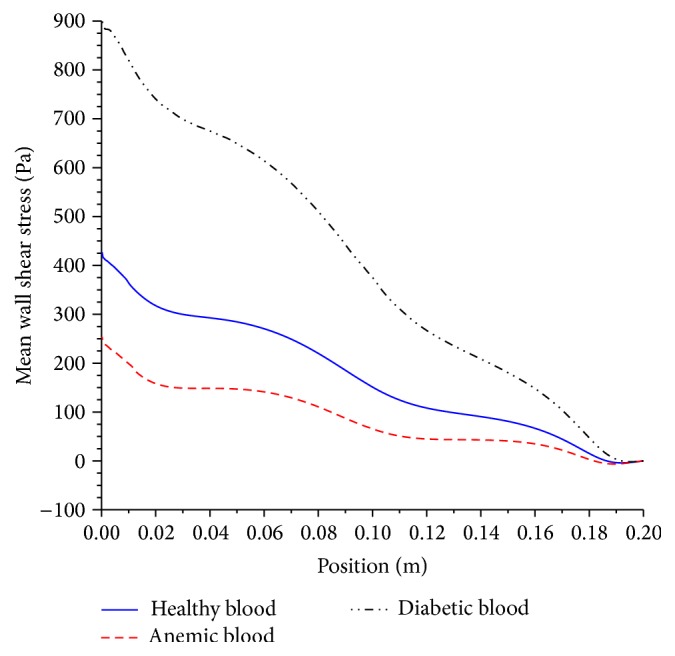
Mean pressure drop. Artery diameter of 5 mm with moderate stenosis, using anemic, diabetic, and healthy blood types.

The greater resistive forces result in higher pressure drop as shown in Figure 18.

**Figure 19:**
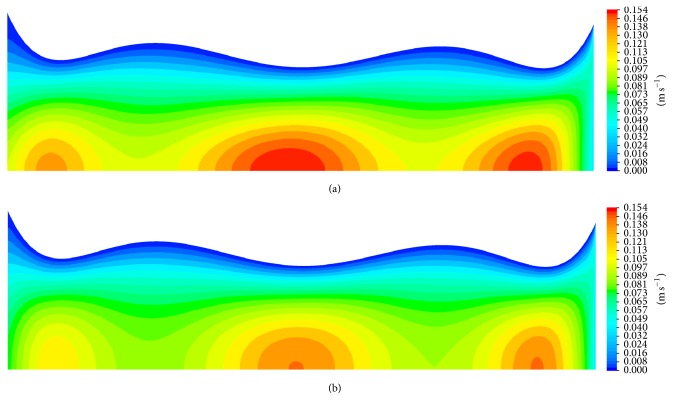
Velocity magnitude contours; artery size, 5 mm; magnetic field, 4 tesla; viscosity, constant viscosity (a) and Carreau viscosity model (b) (radial direction scaled up by a factor of 20 for better illustration).

The action of high local shear stress may worsen stenosed preexisting condition of atherosclerosis lesions and possibly will lead to rupture of plaque or thrombosis which will be mobilized and is likely to occlude in smaller downstream branches forming thromboembolism or blood clot with catastrophic effects. However, anemic blood, which is caused by low red blood cell count, means that the blood acts as Newtonian fluid. The low viscosity of blood leads to lower wall shear stress and pressure drop. Nonetheless, it is also problematic, for the heart, as it is required to work harder to compensate for low count of red blood cell in order to provide adequate supply of needed oxygenated blood. The impact of viscosity and its shear thinning behavior is illustrated by the velocity contours in Figure 19.

## 4. Conclusions

A pulsatile two-dimensional analysis of blood flow with variable viscosity through arteries with multiple stenosis in the presence of transverse magnetic field is studied. The blood flow is assumed laminar, incompressible; both Newtonian and Carreau viscosity models are employed. Moderate and severe stenosis are taken under consideration to offer better understanding of practical problems of blood flow through stenosed artery. Three different types of blood, namely, healthy, diabetic, and anemic are studied.

Using Newtonian viscosity model of blood in contrast to Carreau model is found to underestimate the pressure drop and wall shear stress by nearly by 34% and 40%, respectively. In addition, it is found that using a magnetic field increases the pressure drop by 15%. In addition, doubling the artery diameter would reduce the wall shear stress by approximately 1.6 times. Also increasing the stenosis level from moderate to severe results in reduction of the shear stress by 1.6 times. To nullify the effect of increased level of severity on shear stress the artery size has to be doubled.

The effect of the artery diameter on pressure drop is even greater. Doubling the diameter of moderately stenosis artery results in approximately 3-fold decrease in pressure drop. However, increasing the stenosis level from moderate to the severe results in approximately 3-fold increase in pressure drop. The instantaneous wall shear stress increases with the increase in velocity and local acceleration. In addition, the instantaneous pressure drop increases with increasing velocity and decreases with increasing local acceleration due to favorable pressure gradient in the stenosed sections.

It is also found that a diabetic blood results in higher shear stress and greater pressure drop in comparison to healthy blood, whereas anemic blood has a decreasing effect on both wall shear stress and pressure drop in comparison to healthy blood.

## Figures and Tables

**Table 1:** Experimental data for hematocrit counts and viscosity [27].

Hct	*μ*_*o*_ (Pa·s)	*μ*_∞_ (Pa·s)	*λ* (s)	*n*	Blood
25%	0.0178	0.00257	12.448	0.33	Anemic
45%	0.0161	0.00345	39.418	0.48	Healthy
65%	0.8592	0.00802	103.09	0.39	Diabetic

**Table 2:** Grid independence study using average skin friction factor as a parameter.

Grid size (*z*, *r*)	Artery size (mm)		
	2.5	5	10
	*C*_*f*_		
(110, 20)	0.21875718	0.11173887	0.062403215
(110, 40)	0.21922512	0.11167075	0.061809526
(110, 80)	0.21956481	0.11156201	0.061426839
(110, 110)	0.21939050	0.11174184	0.061336323
(110, 220)	0.21939277	0.11150201	0.061194006

## Nomenclature

*B*: Magnetic flux intensity, tesla
*C*_*f*_: Skin friction coefficient, *C*_*f*_ = *τ*_*w*_/0.5*ρU*_*o*_^2^
*D*_*o*_: Diameter of the artery, m
*E*: Electric field intensity, V/m
*F*_*m*_: Electromotive force, N
*J*: Current density, Amp/m
*L*: Artery length, m^2^
*p*: Pressure, Pa
*r*: Radial coordinate, m
*R*_*o*_: Dimensional radius of the artery, m
*v*_*z*_: Axial velocity, m/s
*U*_*o*_: Average velocity at inlet, m/s
*v*_*r*_: Radial component of velocity, m/s
*V*: Dimensionless radial velocity
*z*: Axial coordinate, m
*α*: Degree of stenosis, %
*λ*: Empirical constant, (9)
*η*: Dynamic viscosity of blood, kg/m s
*μ*_*o*_, *μ*_*∞*_: Asymptotic viscosities equation (9)
*ρ*: Density of blood, Kg/m^3^
*σ*: Electrical conductivity, 1/Ω m
*γ*: Scalar measure of the rate of deformation tensor
*τ*_*w*_: Wall shear stress, Pa·s.
